# The role of Hippo pathway in ovarian development

**DOI:** 10.3389/fphys.2023.1198873

**Published:** 2023-06-02

**Authors:** Mengdi Zhu, Menghao Xu, Jinmin Zhang, Cuihong Zheng

**Affiliations:** Institute of Integrated Traditional Chinese and Western Medicine, Tongji Hospital of Tongji Medical College, Huazhong University of Science and Technology, Wuhan, China

**Keywords:** ovarian development, follicle, Hippo pathway, YAP, follicle activation

## Abstract

The follicle is the functional unit of the ovary, whereby ovarian development is largely dependent on the development of the follicles themselves. The activation, growth, and progression of follicles are modulated by a diverse range of factors, including reproductive endocrine system and multiple signaling pathways. The Hippo pathway exhibits a high degree of evolutionary conservation between both *Drosophila* and mammalian systems, and is recognized for its pivotal role in regulating cellular proliferation, control of organ size, and embryonic development. During the process of follicle development, the components of the Hippo pathway show temporal and spatial variations. Recent clinical studies have shown that ovarian fragmentation can activate follicles. The mechanism is that the mechanical signal of cutting triggers actin polymerization. This process leads to the disruption of the Hippo pathway and subsequently induces the upregulation of downstream CCN and apoptosis inhibitors, thereby promoting follicle development. Thus, the Hippo pathway plays a crucial role in both the activation and development of follicles. In this article, we focused on the development and atresia of follicles and the function of Hippo pathway in these processes. Additionally, the physiological effects of Hippo pathway in follicle activation are also explored.

## 1 Introduction

The ovary is a complex, dynamic structure that undergoes extensive tissue remodeling throughout each reproductive cycle. This periodic remodeling includes follicular formation and atresia, ovulation, formation and regression of the corpus luteum (CL), and accompanying changes in the vascular system and microenvironment, which are essential factors for maintaining normal reproductive function (Brown and Russell, 2014; Vanselow et al., 2020). The ovarian principal role is the periodic generation and release of oocytes, alongside the secretion of steroid hormones (Gougeon, 1996). Within the ovary, the follicle represents the fundamental functional unit, characterized by different types and numbers of cells, which provide the appropriate microenvironment for oocyte development (Gougeon, 1996).

Ovarian reserve refers to the quantity and quality of remaining oocytes in the ovary, which is the basic indicator of female fertility (Practice Committee of the American Society for Reproductive Medicine Azziz et al., 2020). Human follicle development begins at the fourth month of fetal life, peaking at the fifth month of gestation (Markstrom et al., 2002). During the latter stages of embryonic development, the depletion of primordial follicles occurs rapidly through apoptosis. At birth, the total number of primordial follicles ranges between 500,000 and 1,000,000, of which only about 400 will develop into primary oocytes, ovulate and fertilize during the fertile period of the woman, and the majority of the remaining follicles are destined to atresia (Hansen et al., 2008; Findlay et al., 2015).

**FIGURE 1:**
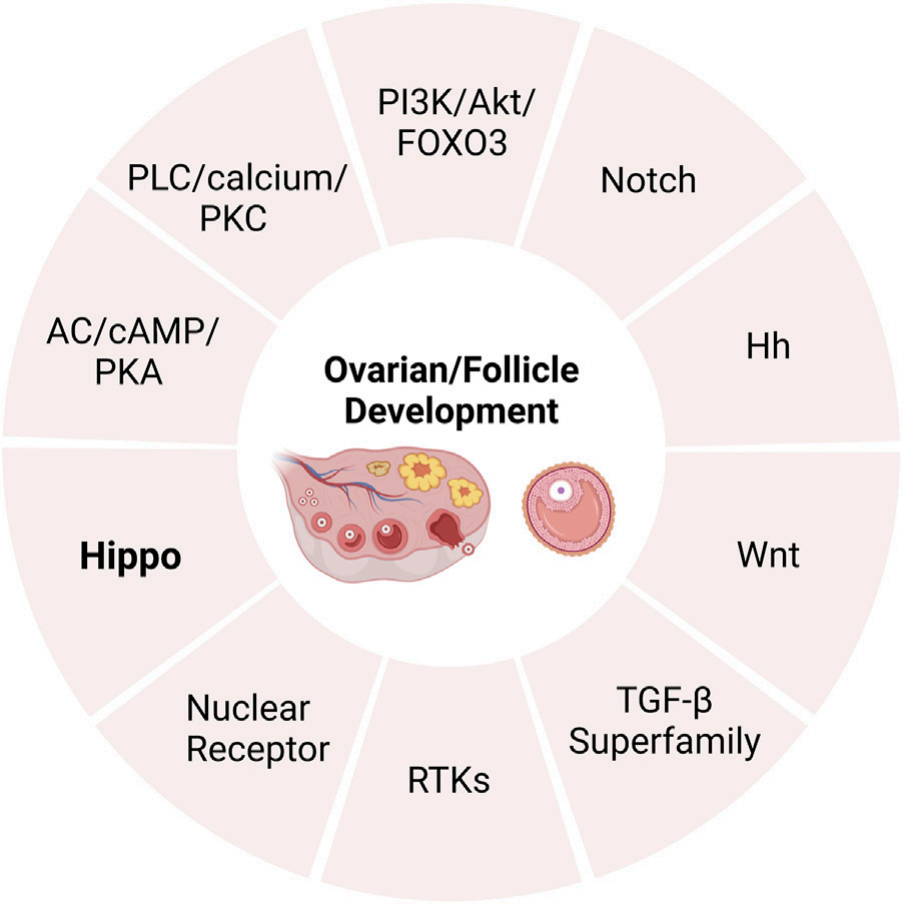
The pathways of regulating ovarian development. AC, adenylate cyclase; PKA, protein kinase A; PLC, phospholipase C; PKC, protein kinase C; TGF-β, transforming growth factor β; RTKs, receptor tyrosine kinases.

Although the endocrine system (consisting of the hypothalamus, pituitary and ovary) exerts a critical influence over follicular development and atresia, more and more pathways are also found to be involved in this regulation (Klionsky, 2008). The intricate interplay of signaling pathways plays a crucial role in the coordinated regulation of ovarian follicular development and subsequent ovulatory processes (Li et al., 2021a). Relevant research has identified several signaling that regulate follicular development, growth, and atresia, including adenylate cyclase (AC)/CAMP/protein kinase A (PKA), phospholipase C (PLC)/calcium/protein kinase C (PKC) (Liu and Ge, 2013), PI3K/Akt/FOXO3 (Zeng et al., 2020), Notch (Xie et al., 2017), Hedgehog (Hh) (Wijgerde et al., 2005; Ren et al., 2012)and Wnt (Castanon et al., 2012; Wang et al., 2013), members of the transforming growth factor β (TGF-β) superfamily (Monsivais et al., 2017), receptor tyrosine kinases (RTKs) (Fan et al., 2009; Chaves et al., 2010) and nuclear receptor (Hughes and Murphy, 2021). In recent years, there has been growing evidence to suggest that the Hippo pathway also plays a critical role in regulating ovarian development (Ma et al., 2019; Dey et al., 2020) (Figure 1). In this article, we explore the role of the Hippo pathway in the complex process of ovarian development.

## 2 Ovarian development

### 2.1 Follicular development

Mammalian oocytes mature in the follicles, which serve as the fundamental units of the ovary. Consequently, ovarian development is fundamentally dependent on follicular development (Li et al., 2021a). Ovarian development begins in the embryonic period, during which it undergoes massive colonization of the ovaries, production of primordial germ cells (PGCs), migration of primordial germ cells to genital ridge, colonization of gonads by PGCs, gonadal sex differentiation, as well as germ cell mitosis and apoptosis (Skinner, 2005; Matsuda-Minehata et al., 2006). After differentiation of PGCs, oocytes begin to develop in the embryo.

**FIGURE 2:**
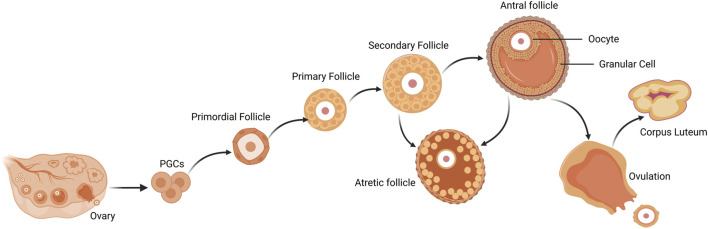
Follicle development and atresia, ovulation of the mammalian ovary. Primordial follicles are activated to become primary follicles after colonization and differentiation of PGCs, which further develop into secondary follicles. Secondary follicles continue to grow into antral follicles. After ovulation, mature follicles degenerate into corpus luteum. During follicular growth and development, most follicles undergo a degenerative process of atresia. PGCs, primordial germ cells.

First, proliferating PGCs migrate to the nascent genital ridge, differentiate into oogonia, which are encapsulated by a single layer of follicular epithelium or granular cells (GCs), and subsequently enter meiosis to become primary oocytes (McLaughlin and McIver, 2009). The primordial follicle is the most basic follicular structure, composed of an oocyte enclosed by a few flattened granulosa cells and basal layer (Hirshfield, 1991). With the regulation of intraovarian growth factors in the ovary and secretion of pituitary gonadotropins, follicles grow a gonadotropin-independent phase to a gonadotropin-dependent phase (Hsueh et al., 2015; Gershon and Dekel, 2020). The transformation of follicles from primordial to primary stage is accompanied by a rapid increase in the cytoplasmic and nuclear volume of oocytes, and changes in the morphology and proliferation of GCs. As follicles develop from primary to secondary stages, GCs around oocytes continue to proliferate and increase in size, and theca cells (TCs) layers begin to form (Hirshfield, 1991). TCs proliferates from undifferentiated mesenchymal cells in ovarian stroma and is a layer of cells close to the basal layer of the follicle (Richards et al., 2018). It is composed of the inner membrane, which contains endocrine cells, and the outer membrane, which is a fibrous connective tissue layer composed of fibroblast-like cells. TCs are the critical component of the ovarian follicle, serving as the source of androgens, Which are subsequently converted into estrogens by GCs (Magoffin, 2005). The TCs layer also contains vascular tissue and immune cells (Young and McNeilly, 2010). As the secondary follicles develop into preantral follicles, the sinus cavity begins to form, the preantral follicles are characterized by multi-layered cube GCs, which undergo continuous proliferation during follicular development, and the TCs layer provides a self-contained blood supply to the growing follicles. In the preantral follicles, TCs exhibit a response to luteinizing hormone (LH), while the differentiated GCs express follicle stimulating hormone (FSH) receptors that facilitate the production and secretion of estradiol (E2) (Edson et al., 2009). GCs of growing antral follicles competitively bind to FSH and grow into dominant follicles. E2 reaches its peak in mature follicles to lead a surge in LH levels, which are necessary for ovulation to occur (de Ziegler et al., 2007). After ovulation, the residual GCs and TCs of the collapsed follicle undergo luteinization and angiogenesis, which forming CL and promoting the secretion of progesterone and other hormones, thereby establishing pregnancy and maintaining early embryonic development (Figure 2).

### 2.2 Generation of oocytes

During follicular growth, the volume of oocytes can increase by 100–300 times and stop at the preantral stage. Furthermore, it has been observed that the quantity of granulosa cells surrounding oocytes exhibits a gradual increase (Keefe et al., 2015), and at this stage, oocytes commence the accumulation of macromolecules such as glycogen, lipid droplets, proteins, and mRNA, in anticipation of their later developmental processes (Sturmey et al., 2009). The oocytes exhibit a high level of synthetic activity, which induces structural changes and the redistribution of the endoplasmic reticulum and Golgi complex. These changes result in the increased number of vesicles and ribosomes and the formation of cortical granules. Additionally, the zona pellucida initiates the secretion of glycoproteins (Picton et al., 1998). Mitochondria are abundant in oocytes and are essential for oocyte maturation, fertilization and subsequent embryogenesis (Kirillova et al., 2021). Mitochondria are unique in that they possess own genetic information except the nucleus. The genetic material in the mitochondria is called mitochondrial deoxyribonucleic acid (mtDNA) (Kukat et al., 2011). Unlike mitochondria in differentiated somatic cells that are mature and form a highly organized network, mitochondria in oocytes display unstructured cristae and limited capacity to generate energy (Trebichalska et al., 2021). During oocyte maturation, the number of mtDNA copy increased significantly. There are two turning points in the rapid increase of mtDNA copies: the initial transition from primordial to primary follicles, and the second transition from the germinal vesicle (GV) stage to the oocyte metaphase II (MII) in antral follicle development. PGCs contain about 200 mtDNA copies, while mature oocytes have about 4 million copies (Santos et al., 2006). At the second turning point of rapid mtDNA copy, a significant change in the distribution of mitochondria occurs, as they move from the center of the ooplasm to the pericortical region and are distributed throughout the ooplasm (Trebichalska et al., 2021). ATP levels also increase significantly as oogenesis progresses (Kirillova et al., 2021). The maturation of oocytes is reliant on the generation of ATP, which is essential for subsequent transcription and translation. Insufficient levels of ATP can cause meiosis errors and result in alterations in chromosome number, culminating in genetic disorders (Dalton and Carroll, 2013).

The maturation of oocytes includes cytoplasmic and nuclear maturation, which must be completed to enable gametes to obtain developmental ability (Eppig, 1996). The nuclear maturation of oocytes involves the following steps: nuclear changes associated with the restoration of meiosis I and the extrusion of the first polar body before MII stops, decondensation of sperm chromatin, formation of the pronucleus, and provision of support for the first division of the embryo. In addition, cytoplasmic maturation during oocyte growth is critical for mRNA post-transcriptional modification, protein synthesis, and regulation of post-translational modification. In the late stage of oocyte development, LH surge promotes the selection and final growth of ovulation follicles, and triggers the expansion of cumulus-oocyte complexes and the loss of gap junctions, eventually leading to oocyte maturation and ovulation. The gradual acquisition of developmental ability during oocyte growth has been well established (Lonergan and Fair, 2016).

It is well known that oocytes not only provide genetic information to developing embryos, but also provide them with energy, nutrients, and mtDNA sets (Kopeika et al., 2015). The differences in embryonic developmental competence can be attributed to changes in mtDNA content, oocytes with low mtDNA copy numbers showing lower developmental potential and thus affecting embryonic viability (Reynier et al., 2001; Van Blerkom, 2011; Ge et al., 2012). Compared with nuclear DNA (nDNA), mtDNA has some special characteristics. It is highly susceptible to the deleterious effects of reactive oxygen species (ROS) and is particularly easy to mutations that lead to functional degradation (Eichenlaub-Ritter et al., 2011). In recent years, oocyte cryopreservation has been widely used in assisted reproductive technology (ART), which can store excess oocytes and preserve fertility for women (Stigliani et al., 2015). At present, slow freezing and vitrification are most commonly used in oocyte cryopreservation (Gardner et al., 2007; Smith et al., 2010). However, slow freezing leads to excessive production of ROS, which affects some key oocyte organelles (such as mitochondria), and mtDNA is damaged by ROS produced within the mitochondrial respiratory chain, further affecting oocyte development and embryo implantation (Ebert et al., 1988; Abedelahi et al., 2008; Kagawa et al., 2009; McCarthy et al., 2010; Farzollahi et al., 2016; May-Panloup et al., 2016). Monzo et al. (2012) examined gene expression profiles in slow-frozen and vitrified oocytes, slow freezing is more detrimental to oocyte developmental competence than vitrification, and it may be the cause of the reduced implantation and pregnancy rates of human oocytes. The indications and frequency of oocyte vitrification in the field of ART are expanding globally (Barberet et al., 2022). It has been demonstrated that the addition of vitrification raises the survival and pregnancy rates of embryo transfer during ART (Cobo et al., 2019). Potdar et al. (2014) found that fresh and vitrified oocytes both responded admirably in terms of fertilization rates, ongoing pregnancy rates, and clinical pregnancy rates. Nottola et al. (2009) further showed that vitrified oocytes generally maintained mitochondrial fine structure after rewarming, with round or oval mitochondria ranging from 0.5 to 0.8 µm in diameter. Regarding the effect of vitrification on genetic integrity and mtDNA carried by oocytes, Ana Arnanz et al. (2020) showed that vitrification of oocytes had no discernible impact on the mtDNA content of trophectoderm biopsy specimens and there was no noticeable distinction in the total rate and mtDNA content between fresh and vitrified blastocysts. It has been reported that the smaller the volume of cryopreservation carrier, the faster the cooling rate, and the higher the cryoprotectant agent concentrations (CPAs), the higher the success rate of vitrification of oocytes (Arav, 2014; Best, 2015). Cobo et al. (2019) found no difference in terms of fertilization rate, day 2 and day 3 cleavage, and blastocyst formation between vitrified and fresh oocytes using the Cryotop method, and the embryo quality was similar on days 3 and 5–6 in vitrified and fresh oocyte groups. Wu et al. (2017) found that LHe vitrification improved the survival of immature bovine oocytes by lessening the deleterious effects of cryodamage on the ultrastructure of certain organelles and the expression of some associated genes as compared to LN vitrification. Zhang et al. (2020) further showed that low vitrification temperatures (VTs) and CPAs could increase blastocyst rate by altering mRNA levels of apoptotic and mitochondrial genes. In conclusion, optimizing the vitrification procedure, setting the appropriate VTs and CPAs, vitrification can better maintain the integrity of oocytes and mtDNA content, which provides a good technical guarantee for the preservation of germ cells during ART.

### 2.3 Atresia of follicles

During follicular development, most follicles undergo a degenerative process of atresia, which makes the primordial follicles continuously depleted (Manabe et al., 2008). Follicular atresia is inhibited by FSH, which is conducive to follicular development. Follicular maturation promotes the secretion of estrogen, which in turn inhibits the secretion of FSH. Follicular atresia is an apoptotic process regulated by threshold-dependent hormones, and the most important part is the apoptosis of GCs (Kaipia and Hsueh, 1997). In the early atretic follicles, apoptotic granulosa cells begin to appear and their number gradually increases. In the progressing atretic follicle, most granulosa cells undergo apoptosis and the granular layer shows severe destruction (Matsuda et al., 2012). Deprivation of critical pro-survival factors or exposure of death ligands are the major causes of apoptosis, both of which lead to the activation of caspase-3, and then participate in the apoptosis of GCs (Matsuda et al., 2012). Follicular atresia is a complex phenomenon involving multiple ligand systems associated with apoptosis, such as FAS ligands, tumor necrosis factor-alpha (TNF-α), and TNF-α-related apoptosis-inducing ligands (Matsuda-Minehata et al., 2006; Quirk et al., 2006; Jaaskelainen et al., 2009). In addition, BCL2 family members regulate germ cell and somatic cell apoptosis by regulating mitochondrial-mediated apoptosis. It plays an important role in follicular atresia (Hsu et al., 1996).

## 3 Hippo pathway and its function

The Hippo pathway, a serine/threonine kinase signaling cascade, is an evolutionary conserved pathway first discovered through screening and identification in *Drosophila melanogaster* (Ma et al., 2019). In mammals, Hippo pathway comprises a set of core components (Avruch et al., 2012), including Mammalian STE20-like kinase 1/2 (MST1/2), Salvador protein 1 (SAV1), Large tumor suppressor kinase 1/2 (LATS1/2), Mps one binder 1A/B (MOB1A/B), yes-associated protein (YAP), transcriptional co-activator with PDZ-binding motif (TAZ) and the transcriptional enhanced associate domain (TEAD) family (Hong et al., 2018).

The activation of the Hippo kinase cascade can be modulated directly or indirectly by multiple upstream signals, including cell polarity, density, stress signals, and soluble factors (Stein et al., 2015). Mitogen activated protein 4 kinase (MAP4K) regulates the activation of MST1/2 and LATS1/2 for phosphorylation and inhibition of YAP/TAZ (Chen et al., 2019). Mechanical stress, such as the stiffness of the extracellular matrix (ECM), are also effective regulatory factors for YAP/TAZ, as the attachment of cells to the tough ECM activates the Rho-GTP enzyme to inhibit the phosphorylation of LATS dependent YAP (Chang et al., 2018). The regulation of the Hippo pathway involves multiple mechanisms, one of which is the activation of G protein-coupled receptors (GPCRs). GPCRs that are coupled to Gα12/13 and Gαq/11, such as lysophosphatidic acid (LPA), sphingosine 1-phosphate (S1P), induce the activation of Rho GTPase, and subsequently increase the levels of filamentous actin (F-actin), which promote the proliferation and migration of epithelial cells on the ovarian surface (Luo and Yu, 2019). Integrin signaling is a crucial factor in regulating the activity of YAP/TAZ in 3D, and it has been found to be associated with differentiation of cultured skeletal stem cells and proliferation of malignant cells (Aragona et al., 2013; Tang et al., 2013). The Hippo pathway is susceptible to inhibition by growth factors such as insulin-like growth factor 1 (IGF1) and epidermal growth factor (EGF) ligand families, which activate RTKs leading to the activation of PI3K/Akt signaling pathway, which further inactivates hippo kinase complex, resulting in increased YAP expression level (Zhou et al., 2020).

**FIGURE 3:**
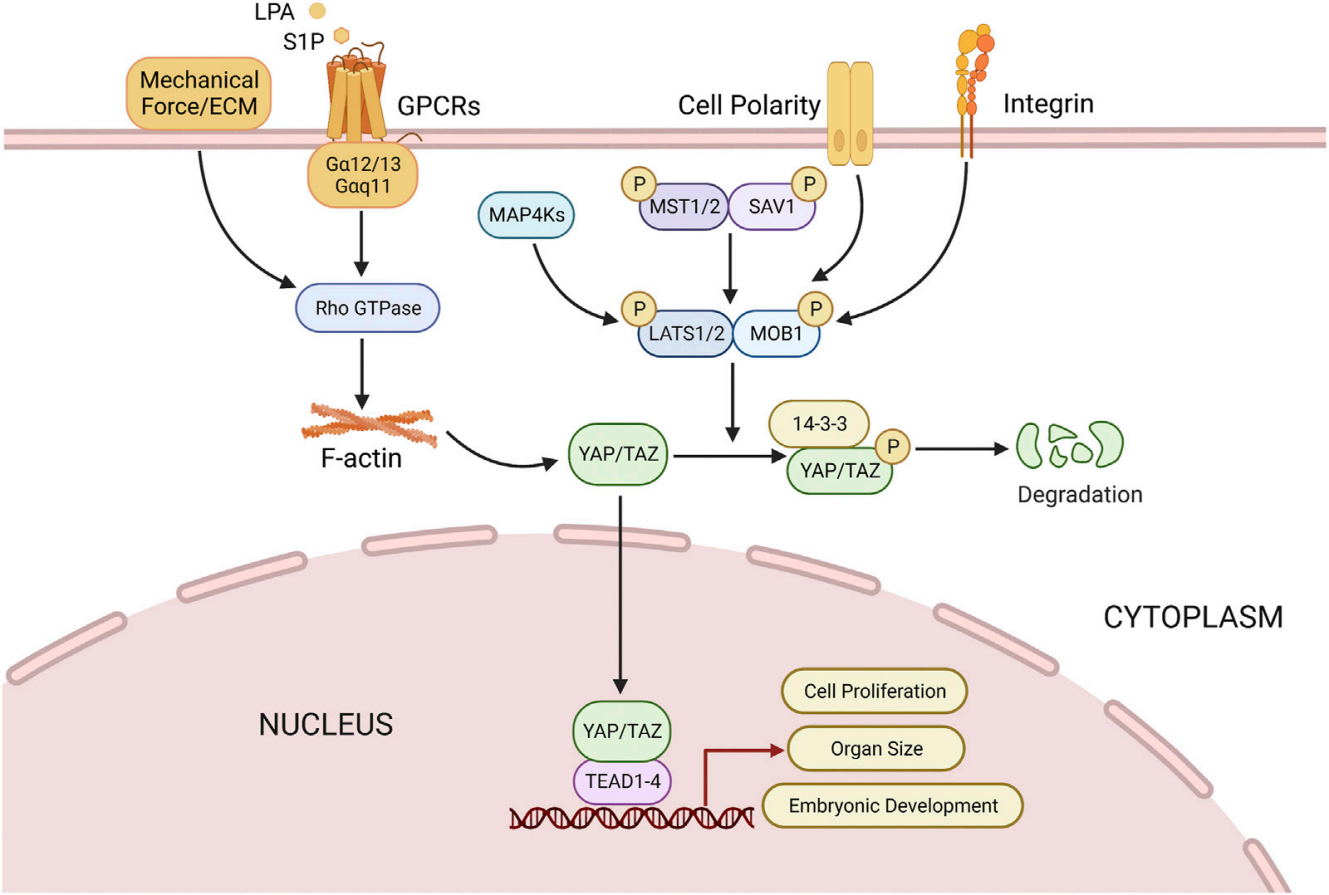
Regulation of Hippo pathway. Multiple factors (Mechanical force, Small molecule soluble substances (LPA, S1P), cell polarity, integrins) can directly or indirectly modulate Hippo pathway, thus regulating cell proliferation, organ size, and embryonic development, etc. See text for details. LPA, lysophosphatidic acid; S1P, sphingosine 1-phosphate.

The activation of the Hippo pathway initiates with the activation of MST1/2 (Chen et al., 2019). MST1/2 forms a heterodimer with SAV1 via its C-terminal SARAH domain, promoting the phosphorylation of SAV1, MOB1 and LATS1/2, as well as the association between MOB1 and LATS1/2 (Karchugina et al., 2021). The phosphorylation of YAP and TAZ at several sites by LATS1/2 is a direct inhibition mechanism for their nuclear translocation (Qi et al., 2022). Further phosphorylation of YAP/TAZ by casein kinase 1 can lead to β-trcp-mediated ubiquitination and proteasome degradation, thereby inhibiting tissue growth and cell proliferation (Liu et al., 2010; Plouffe et al., 2016). Upon the disruption of the Hippo pathway, YAP/TAZ dephosphorylates and translocates into the nucleus to function as a transcription co-activator (Li et al., 2021b; Ito et al., 2021). The binding of YAP/TAZ with transcription factor TEAD facilitates the expression of CCN2/connective tissue growth factor (CTGF) and baculoviral inhibitors of apoptosis repeat containing (BIRC), and then organ size is regulated by cell proliferation, apoptosis and self-renewal of stem cells (Avruch et al., 2012; Clark et al., 2022) (Figure 3).

Hippo pathway is involved in a wide range of biological processes that are mainly mediated by the activity of YAP (Russell and Camargo, 2022). These processes include cell proliferation and differentiation, organ growth, embryonic development, and tissue regeneration (Yu et al., 2015). Disorder of the Hippo pathway has been implicated in various diseases, ranging from cancer to ophthalmic, heart, lung, kidney, liver and ovarian diseases, as well as immune dysfunction (Wang et al., 2020). Therefore, Hippo pathway and YAP protein activity may be the key to the treatment of various diseases (Misra and Irvine, 2018).

## 4 Hippo pathway in ovarian development

The Hippo pathway is involved in regulating essential biological processes like cell proliferation, migration, and differentiation. It also has a critical role in follicle growth and activation, as well as steroidogenesis (Clark et al., 2022). The disorder of Hippo pathway will lead to the loss of follicular homeostasis and reproductive disorders, resulting in a variety of diseases, such as polycystic ovary syndrome (PCOS), premature ovarian failure (POF) and ovarian cancer (Hu et al., 2019a; Siegel et al., 2020; Chang and Dunaif, 2021).

### 4.1 The Hippo pathway regulates follicular development and oogenesis

In mouse and human ovaries, key hippo pathway components (YAP, TAZ, MST1/2, SAV1, and LATS1/2) are expressed in follicles at different stages (Kawamura et al., 2013). Correlational studies have shown that the key components of Hippo pathway, MST1/2, LATS1/2, YAP and phosphorylated YAP (p-YAP), are expressed in oocytes, GCs and TCs from primary to sinus follicular stages, as well as in atretic follicles and corpus luteum (Kawamura et al., 2013; Hu et al., 2019b; Sun et al., 2021). During follicular development, the expression of these molecules in oocytes changes dynamically. De Roo et al. (2020) showed that MST1 progressively translocated from oocyte cytoplasm of primordial and primary follicles to the oocyte nucleus of secondary follicles. In addition, Lv et al. (2020) showed that YAP was located in the nucleus of primary to preovulation follicular granulosa cells, and after ovulation, it is mainly located in the cytoplasm of differentiated corpus luteum cells. However, Sun and Diaz (2019) found that YAP1 was mainly localized in the nucleus in the co-culture of GC and oocytes (Table 1).

**TABLE 1:** Localization of Hippo pathway components during ovarian development.

Study (first author, year)	Model system	Components	Localization
Kawamura et al. (2013)	Mouse model	MST1/2, SAV1, LATS1/2, TAZ	GCs, TCs, oocytes of primordial to secondary follicles
Hu et al. (2019b)	Mouse model	MST1, P-MST, LATS2, YAP1, P-YAP1	The cytoplasm of oocytes and granulosa cells
Sun et al. (2021)	Hen model	LATS2	GCs and oocytes of all the follicles, the ovarian stroma
De Roo et al. (2020)	Ovarian cortex pieces from patients	MST1	Entire oocyte cytoplasm of the primordial, intermediate and primary follicle, clustered around the nucleus in the secondary follicle
Lv et al. (2020)	Primary human granulosa cells	YAP1	The nuclei of GCs in the growing follicle and cytoplasm of luteinized GCs in corpus luteum
Sun and Diaz (2019)	Co-culture of GC and oocytes *in vitro*	YAP1	The nuclei of GCs and oocytes

MST1/2, Mammalian STE20-like kinase 1/2; SAV1, Salvador protein 1; LATS1/2, Large tumor suppressor kinase 1/2; TAZ, transcriptional co-activator with PDZ-binding motif; GCs, granular cells; TCs, theca cells; YAP, yes-associated protein.

The upstream negative regulators of the Hippo pathway, including MST1/2, SAV1 and LATS1/2, have been shown to regulate the growth, development and function of the mammalian ovary (Tsoi et al., 2019). At present, the role of Hippo pathway component MST1/2 in follicular development is less studied (Lyu et al., 2016). Studies utilizing avian follicles as models have demonstrated that mRNA expression levels of SAV1 are highest in small follicles (<1 mm), and decreased with the development of follicles. Silencing the *Sav1* gene by small interfering RNA (siRNA) can disrupt the Hippo pathway, leading to elevated YAP activity and subsequently promote proliferation of granulosa cells (Lyu et al., 2016). LATS1/2 is an important regulatory factor of the Hippo pathway. Sun et al. (2021) observed the regulatory role of LATS2 in the development of chicken ovary and found that LATS2 was mainly located in oocytes and undifferentiated GC of pre-layered follicles of chicken ovary, and the expression of *Lats2* mRNA in smaller follicles and GC was significantly higher than that in larger follicles. High expression of LATS2 inhibits GC proliferation and differentiation, follicle selection, and mRNA and protein expression of biomarker related genes for steroid-production, including FSH receptor (FSHR), steroidogenic acute regulatory protein (STAR), estrogen receptor (ESR), etc. (Sun et al., 2021). At the same time, Silencing the *Lats* gene by siRNA can lead to enhanced proliferation of granulosa cells and increased expressions of FSHR, STAR and ESR (Vanselow et al., 2020).

YAP is an effector molecule downstream of Hippo pathway and plays a pivotal role in ovarian development (De Roo et al., 2017). Hu et al. (2019b) found that the expression of the core components of Hippo pathway changed with follicle development by observing the expression of the major components of Hippo pathway in the ovaries of mice at 3 days, 1, 5, and 16 months postnatally. The expression of MST1 and LATS2 decreased significantly with increasing age of the mice, while the expression levels of YAP1 and p-YAP showed the opposite trend (Hu et al., 2019b). It has been observed that the expression of YAP in mouse ovarian germ cells during embryonic development is negligible at 13.5 days of gestation but increases gradually in pups at 15.5, 18.5, and 2 days of gestation (Abbassi et al., 2016). All these results suggested that the expression of YAP in mouse ovary increases with the growth of follicles (Hu et al., 2019b; Abbassi et al., 2016). In the cultured mouse ovary *in vitro*, the low expression of *yap* inhibited follicle growth, whereas overexpression of *Yap* resulted in follicle activation as indicated by decreased primordial follicle number and increased secondary follicle number (Hu et al., 2019b; Xiang et al., 2015; Lv et al., 2019). These studies have shown that YAP1 mainly induces the transition of follicles from primordial to primary stages (Gershon and Dekel, 2020). A recent investigation demonstrated that the conditional knockout of the *Yap* gene in granulosa cells of mice resulted in abnormal ovarian follicle development, decreased ovarian volume, augmented follicular atresia, as well as reduced litter size and number of offspring (Lv et al., 2019). The overexpression of *Yap* also increased the thickness of ovarian surface epithelium in chemotherapy-induced infertile mice, promoted follicular recruitment and increased the live birth rate (Devos et al., 2020). Therefore, the Hippo pathway effector YAP plays an important role in follicular development and function.

The appropriate growth and maturation of oocytes is essential for successful reproduction in mammals, and these processes are dynamically coupled (Shah et al., 2018). YAP1 has been discovered in mature oocytes using single-cell transcriptome analysis of oocytes taken from *in vitro* fertilization patients (Yan et al., 2013). YAP1 is not intrinsically involved in mouse oogenesis (Abbassi et al., 2016). Oocyte-specific deletion of *Yap1* had no effect on folliculogenesis, oocyte maturation, spindle organization or fertilization (Yu et al., 2016). Sun and Diaz (2019) found that prior to ovulation, oocytes inhibit the Hippo pathway to activate YAP1 and increase granulosa cell survival and proliferation, while inhibiting cell differentiation, and these effects are reversed by ovulation signaling, which first inhibits YAP1 and then leads to YAP1 degradation, thereby promoting cell differentiation. Nuclear YAP has no significant physiological role during mammalian oocyte development (Abbassi et al., 2016). Multiple mechanisms synergistically act to prevent YAP accumulation in the oocyte nucleus (Basu et al., 2003). PKA can phosphorylate LATS kinase and promote S112 to phosphorylate YAP to anchor it in the cytoplasm (Abbassi et al., 2016). It is clear that the Hippo signaling pathway controls the development of follicles and the maturation of oocytes in ovarian somatic cells as opposed to germ cells (Clark et al., 2022). However, oocyte *Yap1* is essential for embryonic development after fertilization (Sha et al., 2020). The abundance of F-actin bundles in the trophectoderm of early mammalian embryos is linked to higher YAP activity and cell proliferation (Slager et al., 1992).

During oogenesis, the oocyte is responsible for establishing the polarity of the egg and embryo along the anterior-posterior (AP) and dorsal-ventral axes, and follicular cell differentiation along the AP axis is a critical step in the normal development of the egg chamber and the establishment of oocyte polarity (Wong et al., 2015). Yu et al. (2008) showed that the posterior follicle cell (PFC) of *Drosophila* require the Hippo pathway and its downstream target Yorkie (Yki) to form AP axis polarity of oocyte compartment. Several signaling pathways in the polar follicle cells (PFCs) are essential for the establishment of dorsal-ventral polarity during mid-oogenesis. These pathways include JAK/STAT, EGFR, Notch, and Hippo (Wong et al., 2015). One of the fundamental events during oocyte maturation and meiosis is cell polarization (Pennarossa et al., 2021). In non-polarized cells, angiomotin (AMOT) is phosphorylated and activates NF2 and LATS1/2 kinases, promoting YAP/TAZ phosphorylation and inducing their cytoplasmic retention (Zhao et al., 2011; Li et al., 2015a). In contrast, in polarized cells, AMOT is sequestered by the PAR-aPKC system, leading to dephosphorylation of YAP/TAZ, with consequent nuclear accumulation (Hirate and Sasaki, 2014).

### 4.2 Effect of hippo pathway on ovarian endocrine secretion

The abnormality of Hippo pathway leads to the abnormal secretion of ovarian gonadotropin levels and other hormones. Related studies revealed that deletion of Lats1 in female mice led to increased ovarian weight, loss of granulosa cell function, and reduced serum levels of LH, prolactin, and progesterone, while FSH levels were not affected (Tsoi et al., 2019; Menard et al., 2020). Hormone-dependent E2 production is critical for the proper functioning of granulosa cell and contributes to the regulation of the reproductive cycle (Chauvin et al., 2022). A study utilizing siRNA to silence the *Yap1* gene in bovine granulosa cells *in vitro* has demonstrated a marked decrease in FSH-induced E2 production, with an 80% reduction observed in comparison to the control group (Plewes et al., 2019). Similarly, the stimulation with testosterone and E2 was observed to elevate the expression and function of YAP (Ji et al., 2017).

LH and FSH are heterodimeric glycoproteins that share a common α-subunit and differ in their respective β-subunits [LHβ (Lhb) and FSHβ (Fshb)]. The transcription of Fshb is mainly regulated by TGFβ superfamily ligands, the most prominent of which is activin (Kumar, 2018). Activin binding to FSHR promotes intracellular phosphorylation of SMAD2 and SMAD3 proteins, which form a complex with SMAD4 and translocate to the nucleus. In combination with forkhead box L2, the SMAD2/3/4 complex functions as a transcription factor to promote the transcription of Fshb (Li et al., 2017). Related studies showed that the Hippo pathway interacted with the function of TGFβ/activin of receptor-regulated SMAD, and in epithelial cells, high cell density promoted the phosphorylation of YAP/TAZ and binding to the TGFβ-induced SMAD2/3/4 complex, which led to its cytoplasmic segregation and inhibition of TGFβ signaling (Grannas et al., 2015). Furthermore, the SMAD complex can regulate the transcription of Lhb, Ariane et al. showed that YAP/TAZ act as inhibitors of basal gonadotropin secretion (Lalonde-Larue et al., 2022). YAP/TAZ play a vital role in the regulation of Fshb and Lhb transcription.

The Hippo and ERK pathways may potentially converge in the regulation of ovarian function (Fan et al., 2009). Ji et al. (2017) observed an increase in the expression of phosphorylated MST and a decrease in the expression of YAP1 in granulosa cells of ERK1/2-deficient superovulatory mouse model, while no significant changes in the expression of YAP1 were observed. In various cell types, MAPKs co-regulate LATS1/2 with MST1/2 (Meng et al., 2015). Pre-ovulatory LH surge leads to activation of the RAS-ERK1/2 signaling cascade in the ovary. RAS signaling is critical for the inactivation of the Hippo pathway, and it has been shown that EGFR-RAS-RAF-MEK-ERK-mediated interactions can regulate the Hippo pathway (Zinatizadeh et al., 2021). *In vitro* treatment of bovine granulosa cells with vetiporfin, a YAP1/TEAD inhibitor, the downregulation of CTGF downstream of YAP1 and a decrease in epidermal growth factor receptor mRNA abundance were observed (Dos Santos et al., 2022). The Hippo pathway plays a critical role in regulating cell differentiation and thus regulates the onset of gonadotropin-dependent ovulatory response (Clark et al., 2022).

### 4.3 Application of disrupting hippo pathway in follicular activation

**FIGURE 4:**
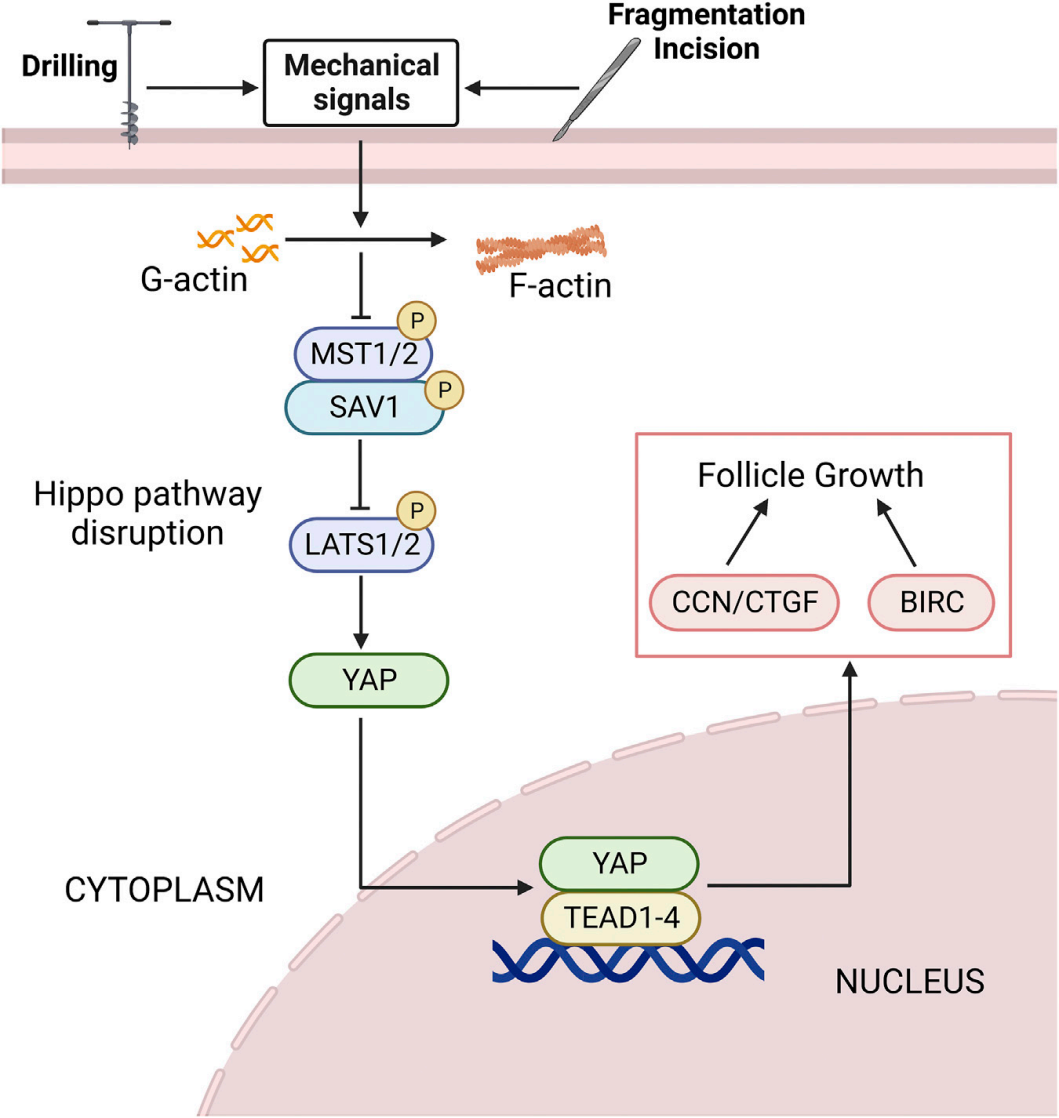
The Hippo pathway is disrupted by mechanical signaling. Mechanical signals such as ovarian fragmentation and incision, drilling can promote actin polymerization, thereby disrupting the Hippo pathway. The expression level of upstream negative regulators of the Hippo pathway is decreased, and the nuclear localization of YAP is increased. It binds to the TEAD1-4 to promote the expression of downstream CCN/CTGF and BIRC, thereby promoting follicle growth and development. See text for details. YAP, yes-associated protein; TEAD, transcriptional enhanced associate domain; CTGF, connective tissue growth factor; BIRC, baculoviral inhibitors of apoptosis repeat containing; G-actin, global-actin; F-actin, filamentous actin.

Mechanical signals from ECM, cell adhesion sites and shape, and actin cytoskeleton can regulate Hippo pathway, thereby regulating cell proliferation and differentiation (Dasgupta and McCollum, 2019) (Figure 4). Cultivating mesenchymal stem cells (MSCs) in a high hardness ECM can increase the activity of YAP and promote bone formation, while cultivating in a soft matrix can reduce the activity of YAP and promote adipogenesis (Mohri et al., 2017). Recent clinical studies have shown that ovarian cortical tissue fragmentation followed by autologous transplantation can activate follicles, increase serum E2 levels in patients with decreased ovarian reserve function, and thereby improve fertility (Fabregues et al., 2018; Lunding et al., 2019). In the ovary, mechanical stimuli such as fragmentation, incision, drilling, or wedge resection lead to actin polymerization, thereby blocking Hippo signal transduction. Studies have shown that F-actin formation in stress fibers is necessary for Hippo pathway blockade and YAP nuclear localization (Wada et al., 2011). After follicular fragmentation, YAP is predominantly expressed in the nuclei of oocytes and GCs in ovarian wedge sections. The nuclear YAP interacts with TEAD transcription factors, leading to increased expression of downstream CTGF and BIRC, which ultimately promotes the proliferation of granulosa and membrane cells, thus facilitating follicular growth (Hsueh and Kawamura, 2020). In addition, coculture of broken ovaries with protein kinase B (PKB/AKT) activators can promote the growth of secondary and sinus follicles. In contrast, the use of AKT inhibitor MK2206 can inhibit follicular growth, and overexpression of *Yap1* can partially offset the inhibition of follicular growth caused by MK2206 (Hu et al., 2019b).

Extensive studies have shown that disrupting the Hippo pathway by mechanical signaling can activate follicles (Table 2). Cheng et al. (2015) treated mouse ovaries with actin polymerization agent S1P, and after treatment, it was found that F-actin expression was increased, and the expression of YAP and CTGF in Hippo pathway nucleus was increased, and short-term treatment and retransplantation of ovaries with S1P *in vitro* can promote follicular growth, Pors et al. (2020) confirmed this result later. Hu et al. (2019b) cultured the ovaries of mouse pups *in vitro* and detected the changes in the components of Hippo pathway during culture. The findings demonstrated that Hippo-YAP1 signaling pathway played a crucial role in the activation of primordial follicles in mice. Devos et al. (2020) cut and cultured ovaries and found that ovarian fragmentation disrupted the Hippo pathway and promoted YAP recruitment in oocytes and GCs nuclei. Kawamura et al. (2013) conducted allogeneic transplantation after ovarian rupture in mice, and found that the expression of F-actin increased, resulting in the blocking of Hippo pathway. They observed a decrease in phosphorylated YAP, an increase in nuclear localization of YAP, and an increase in downstream growth factors and apoptosis inhibitors, thereby promoting follicular growth and oocyte maturation. Grosbois and Demeestere (2018); Masciangelo et al. (2020) further cultured frozen-thawed ovarian cortical fragments from clinical patients *in vitro* and found that ovarian fragmentation resulted in the translocation of Hippo effector YAP to the granulosa nucleus, promoted the upregulation of downstream targets BIRC1 and CCN2, and led to increased follicle growth near the incision site. De Roo C et al. presented evidence of Hippo pathway involvement in primordial follicle activation *in vitro*, specifically during the period from day 0 to day 4 (De Roo et al., 2020). Therefore, blocking the Hippo pathway is an important link in follicular activation, and ovarian tissue rupture and retransplantation can activate follicles. This discovery provides ideas for the clinical treatment of early-onset ovarian insufficiency and POF (Lunding et al., 2019). However, Lunding et al. (2020) found that actin polymerization, the expression of YAP and downstream CCN2 were not increased after *in vitro* fragmentation of frozen ovarian cortex, suggesting that fragmentation is likely to be ineffective to activate follicle growth in human ovarian cortex. This indicated that the effect of Hippo pathway in the process of follicle activation may be spatiotemporal and thus difficult to detect its changes with fewer investigation timepoints. More studies are needed to verify the regulatory role of F-actin and Hippo pathway in human ovarian tissue fragmentation.

**TABLE 2:** Activation of follicles by disrupting Hippo pathway through mechanical signals.

Study (first author, year)	Model system	Methods	Findings
Cheng et al. (2015)	S127A mutation female mice with CD-1 and SCID	Paired ovaries from day 10 mice treated with JASP and S1P	JASP and S1P induced actin polymerization increased; led to nuclear localization of YAP, CCN2 expression, and follicle growth
Pors et al. (2020)	Mouse model; human ovarian tissue	Incubation of murine ovaries with S1P; incubation of human ovarian cortex with S1P and xenotransplantation	S1P increased Ccn2/CCN2 gene expression in isolated preantral follicles and ovarian tissue from mice and human
Hu et al. (2019b)	Mouse model	Ovaries of female pups were cultured *in vitro*	Primordial follicle activation was accompanied with the attenuation of the Hippo pathway
Devos et al. (2020)	Immature ovary of the mouse	Cut the ovaries in half before culture	Fragmentation induced follicle activation through both the PI3K and Hippo signaling pathways
Kawamura et al. (2013)	Mouse model	Fragmented ovaries juvenile (Day 10) mice and allo-transplantation	Decreased p-YAP levels; increased nuclear localization of YAP; secreted CCN2 promoted follicle growth after transplantation
Grosbois and Demeestere (2018)	Frozen–thawed ovarian cortex fragments of patients	Ovarian fragments were analyzed at Days 0, 1, 3 and 5 of culture	Promoted translocation of the YAP into the nucleus of granulosa cells, led to upregulation of BIRC1 and CCN2; increased the follicular growth near the cutting site
Masciangelo et al. (2020)	Frozen-thawed ovarian tissue from women	Obtain 4 fragments of ovarian tissue; one to be non-grafted and three to be grafted	Growing follicles increased; primordial follicles expressing YAP increased
De Roo et al. (2020)	Ovarian cortex pieces from patients	Cortical strips of ovaries were cultured *in vitro*	Primordial follicles were activated *in vitro* by disrupting Hippo pathway, predominantly from Day 0 to Day 4
Lunding et al. (2020)	Frozen ovarian cortex from women	Fragmentation of the ovarian cortex *in vitro*	Fragmentation is likely to be ineffective to disrupt Hippo pathway and activate follicle growth

YAP, yes-associated protein; SCID, severe combined immunodeficient; JASP, Jasplakinolide; S1P, sphingosine-1-phosphate; hUCMSC-Exos, human umbilical cord mesenchymal stem cell-derived exosomes; BIRC, baculoviral inhibitors of apoptosis repeat containing.

### 4.4 Hippo regulates ovarian development by influencing other pathways

It has been reported that several coordinated signaling pathways are interconnected to regulate ovarian folliculogenesis, starting with the dormant primordial follicle and ending with the fully mature and normally formed oocyte ready for fertilization (Jones and Shikanov, 2019). As the main effectors of the Hippo pathway, YAP1/TAZ are crucial for granulosa cell proliferation and follicle development, and the specific loss of YAP in GCs increases cell apoptosis and leads to subfertility (Hsueh and Kawamura, 2020). YAP1/TAZ are involved in the regulation of several signaling pathways including PI3K/Akt, Wnt, TGFβ, Hh, Notch, and IGF, and act in concert to control follicular development (Vanuytsel et al., 2013).

PI3K/Akt/mTOR is a pathway regulated by YAP that regulates cell size, tissue growth and proliferation (John et al., 2008). Activation of the PI3K/AKT pathway can lead to FOXO3 phosphorylation and nuclear export, further inducing primordial follicle activation, which results in rapid depletion of the follicle pool and subfertility (Reddy et al., 2010; Adhikari et al., 2012). In mammalian ovary, FOXO3 is located in GCs at all stages of follicular development and plays a designated role in proliferation, apoptosis and differentiation of them, as well as oocyte maturation, maintaining primordial follicle quiescence, thereby inhibiting follicle activation to increase ovarian reserve (Castrillon et al., 2003; Cunningham et al., 2004). Devos et al. (2020) confirmed that *in vitro* follicle activation is mainly involved in PI3K/AKT/mTOR and Hippo pathways, and both can be regulated by mTORC1 inhibitors, indicating a link between the two pathways. Loss of Phosphatase and tensin homolog (PTEN) in mice promotes the expression of Akt and/or mTOR (Tanaka et al., 2012). In human gastric cancer cell lines, PTEN knockdown increased the nuclear localization of YAP (Xu et al., 2018). The ability of Everolimus acting downstream of AKT to prevent 4-hydroperoxycyclophosphamide-induced Ccn2 expression suggests an alternative connection between the two pathways, and the specific mechanism is not clear but it has been shown that mTORC2 may directly activate Rho protein, a small G protein that regulates cell shape by stimulating actin polymerization into filaments and ultimately disrupting the Hippo pathway (Makker et al., 2014). In addition, Borreguero-Munoz et al. (2019) suggested that follicle cells of *drosophila* must receive an insulin/IGF-1 signal that activates PI3K-PDK1-Akt to inhibit Hippo kinase, possibly through Akt phosphorylation or other mechanisms to be elucidated. LNK is an important regulator of insulin signaling pathway and can promote granulosa cell apoptosis in PCOS by negatively regulating the insulin-stimulated PI3K/AKT/FOXO3 pathway (Luo, 2017). Pre-follicular development is significantly initiated when PTEN inhibitors or PI3K activators are added to human ovarian tissue (McLaughlin et al., 2014; Novella-Maestre et al., 2015). The interaction of Hippo and Akt has been used for *in vitro* activation (IVA) of follicles, ovarian fragmentation (Hippo signaling disruption) followed by IVA drug treatment (AKT stimulation). Successful pregnancies have been reported after transplantation of small ovarian fragments exposed to PI3K/Akt activators into patients diagnosed with POF (Kawamura et al., 2013; Suzuki et al., 2015).

**FIGURE 5:**
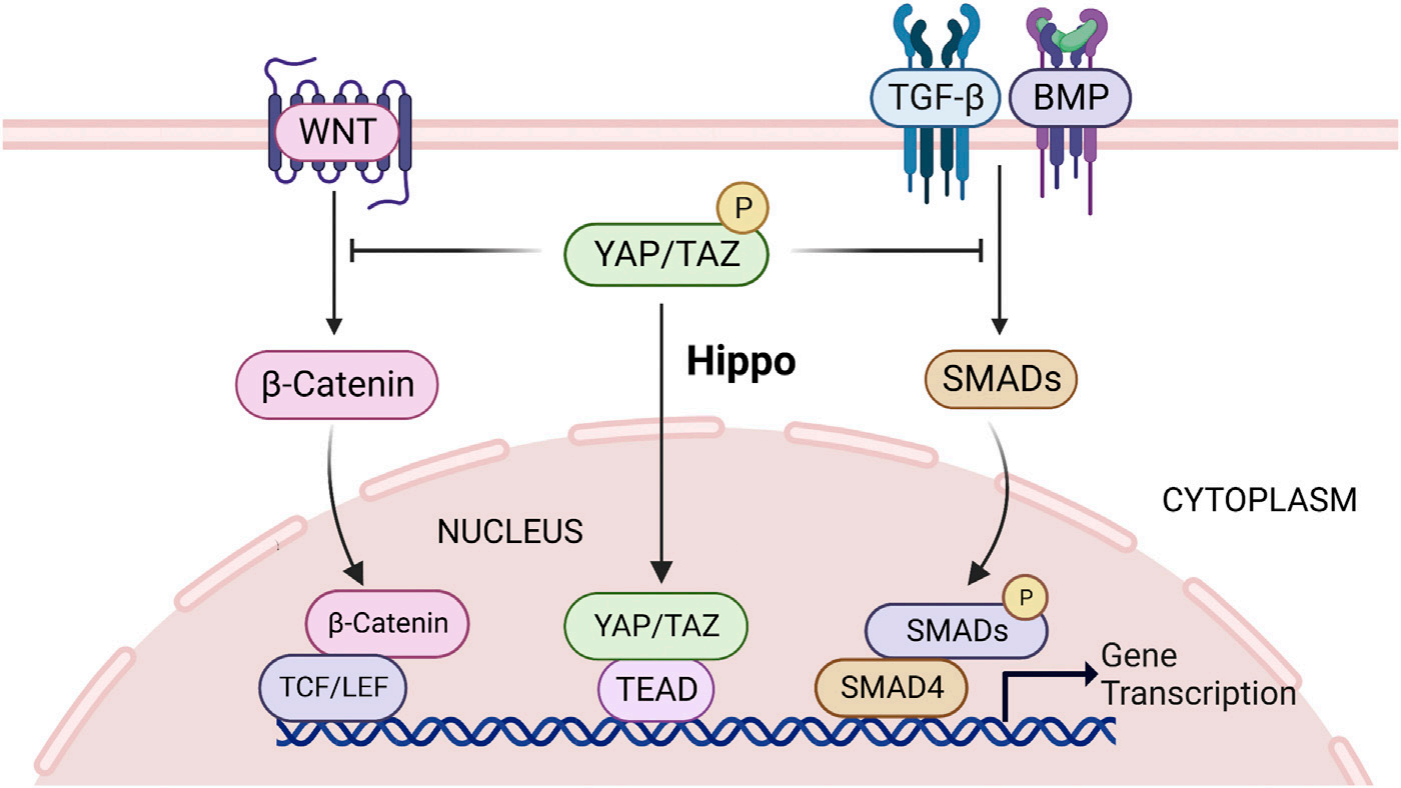
Hippo signaling can interect with Wnt/β-catenin and TGF-β signaling, and its regulators YAP/TAZ locate at the crosstalk between Wnt/β-catenin and TGF-β/Smads. When the Hippo pathway is activated, YAP/TAZ is sequestered in the cytoplasm, thereby inhibiting Wnt/β-catenin and TGF-β activity and subsequent gene transcription. See text for details. TGF-β, transforming growth factor β; YAP, yes-associated protein; TAZ, transcriptional co-activator with PDZ-binding motif.

The canonical Wnt/β-catenin signaling pathway is a cellular communication system that plays a pivotal role during embryogenesis (Steinhart and Angers, 2018). TGF-β cytokine family, such as TGF-β, bone morphogenetic protein (BMP), and activin, which can regulate a variety of biological activities in various cell types and at different developmental stages (Luo, 2017). Hippo signaling can interect with Wnt/β-catenin and TGF-β signaling to control cell growth, and its regulators YAP/TAZ locate at the crosstalk between Wnt/β-catenin and TGF-β/Smads, which provides the transcriptional response, and these responses converge on the transcription of target genes that function in the nucleus (Attisano and Wrana, 2013). YAP/TAZ is trapped in the cytoplasm while the Hippo pathway is active, which prevents Wnt/β-catenin and TGF signals (Attisano and Wrana, 2013) (Figure 5). Similarly, Varelas et al. (2010) also showed that cytosolic TAZ is a negative regulator of the Wnt pathway, and elimination of LATS expression leads to nuclear accumulation of TAZ, reduced TAZ-Dishevelled (DVL) interaction, enhanced DVL phosphorylation, and promotes Wnt pathway activation in cells, ultimately leading to elevated β-catenin levels. β-catenin is a crucial molecule in the Wnt pathway (Li et al., 2021a). In the mammalian intestinal canal, an increase expression of YAP1 induces nuclear β-catenin accumulation, thereby activating the Wnt signal and maintaining crypt stem cell proliferation and stemness (Camargo et al., 2007; Zhou et al., 2011). Previous studies have also shown that β-catenin activation can promote FSH-mediated effects in ovarian follicle cells (Wang et al., 2010; Hernandez Gifford, 2015). Wang et al. (2013) showed that WNT2 regulates DNA synthesis in mouse granule cells via β-catenin (Wang et al., 2013). In addition, overexpression of *Wnt2* in GCs promoted cell proliferation and increased β-catenin levels in the cytoplasm and nucleus (Wang et al., 2010). YAP/TAZ can also interact with TGF-β regulated Smads, interfering with hippo activity of the cell results in concomitant nuclear accumulation of YAP/TAZ and Smads (Varelas et al., 2008). Subsequently, TAZ/YAP cooperates with Smads to promote the activation of specific target genes that control the maintenance of stem cell pluripotency or the induction of differentiation (Varelas et al., 2008). In addition, YAP/TAZ-TEAD and SMAD may act synergistically to promote follicle growth by disrupting the Hippo pathway *in vitro* (Grosbois and Demeestere, 2018). GDF9 and BMP15, the members of the TGFβ superfamily, which are expressed in oocytes during most of folliculogenesis. They are participated in specific functions of GCs and cumulus cells (McGrath et al., 1995; Laitinen et al., 1998; Gilchrist et al., 2008). During follicular growth, they activate the canonical TGF-β signaling cascade in the GCs of growing follicles where CCN2 mRNA is abundantly expressed (Harlow and Hillier, 2002). Alarcon et al. (2009) found that in embryonic stem cells of mouse, phosphorylated SMAD1-SMAD4 complexes recruit YAP1 to the enhancer regions of BMP-responsive genes, leading to BMP-induced gene expression and further induction of cell differentiation. In conclusion, the reduction of cytosolic YAP/TAZ after Hippo pathway disrupted could activate WNT and TGFβ signaling pathways to jointly promote granulosa cell proliferation and follicular development.

There are few studies investigating the mechanism of Hippo and Hh interaction in the human ovary, and there is evidence for an association between the Hippo and Hh pathways in medulloblastoma (Hong et al., 2016). Activation of Sonic hedgehog of cerebellar granule neuron precursors upregulates YAP1 expresssion, and immediate binding between YAP1 and insulin receptor substrate 1 (IRS1), leads to the nuclear accumulation of YAP1 (Fernandez et al., 2009). Hh signaling in *Drosophila melanogaster* ovarian follicle stem cells (FSCs) increases the activity of Yki. Both Hh signaling and Yki positively regulate the rate of FSC proliferation (Huang and Kalderon, 2014). Li et al. (2015b) further demonstrated in *Drosophila* ovaries that Cubitus interruptus (Hh signaling effector) inhibits Hippo activity, which impairs the formation of the Hpo-Wts signaling complex, thus increasing the expression of Yki in nucleus.

Regarding the association of Hippo and Notch pathway, related studies have found that in the intestinal canal, activated YAP1 transcription upregulates Notch receptor expression, leading to an increase of Notch signaling activity (Hong et al., 2016). Several members of the Notch pathway, such as Notch1 and Notch2, were upregulated in hepatocytes with *Yap1* overexpression, and Notch2, identified as the targeted gene of YAP1-TEAD complex, was expressed at high levels in the cell membrane and GCs of ovarian follicles (Yimlamai et al., 2014; Jing et al., 2017). Meignin et al. (2007) showed that the Salvador-Warts-Hippo (SWH) Pathway is required for Notch signaling in *Drosophila* ovaries and that the SWH signal is involved in Notch-dependent maturation of the PFC.

The mechanism of Hippo pathway and the crosstalk of several pathways to regulate ovarian development is complex and diverse, so a large number of experimental studies are still needed to confirm.

## 5 Conclusion

Hippo pathway and its downstream transcription effector YAP/TAZ have emerged as key regulatory players in ovarian development and function, regulating the proliferation and migration of ovarian germ cells and somatic cells, follicular activation and development, and cell apoptosis. This article elucidates the process of ovarian development and the function and molecular mechanism of Hippo pathway in it. However, ovarian development and regulatory mechanisms are complex, and Hippo pathway intersects with other pathways that regulate follicular development, and is affected by ovarian endocrine and metabolic regulation. Therefore, further experimental research is still needed to clarify the localization and regulatory mechanism of Hippo pathway molecules at various stages of follicular development, which is of great significance for clinical treatment of various ovarian diseases.
